# Outcomes in Early Adulthood for Individuals Born Very Preterm and/or with Very Low Birth Weight: Evidence from Multinational Cohorts

**DOI:** 10.1016/j.jpedcp.2025.200196

**Published:** 2025-12-01

**Authors:** Hanifa Pilvar, Catia Nicodemo, Stavros Petrou, Brian A. Darlow, Paula van Dommelen, Kari Anne I Evensen, Sarah Harris, John Horwood, Karen Mathewson, Saroj Saigal, Louis Schmidt, Dieter Wolke, Lianne J. Woodward, Sungwook Kim

**Affiliations:** 1 Nuffield Department of Primary Care Health Sciences, University of Oxford, Oxford, United Kingdom; 2 Brunel University of London, Uxbridge, England; 3 University of Otago, Otago, New Zealand; 4 The Netherlands Organization for Applied Scientific Research (TNO), Leiden, the Netherlands; 5 Norwegian University of Science and Technology, Trondheim, Norway; 6 McMaster University, Hamilton, Ontario, Canada; 7 University of Warwick, Coventry, United Kingdom; 8 University of Canterbury, Christchurch, New Zealand

**Keywords:** educational attainment, long-run outcomes, preterm birth, RECAP project

## Abstract

**Background:**

Advances in neonatal care have improved survival rates for infants born very preterm (VP) and/or with very low birth weight (VLBW), yet their long-term outcomes into adulthood remain understudied.

**Objectives:**

To assess the impact of VP/VLBW status on mortality, educational attainment, and labor market outcomes in early adulthood using data from the RECAP Preterm Project.

**Methods:**

We used harmonized data from 5 nationally representative cohort studies in high-income countries (Canada, Germany, the Netherlands, New Zealand, and Norway) participating in the RECAP Preterm Project. Our sample included 2493 individuals born VP/VLBW and 496 control patients born at term. We used coarsened exact matching to compare adult outcomes between infants who were VP/VLBW and those born at term and an instrumental variable approach—using maternal nulliparity—to estimate the marginal effect of gestational age within the VP/VLBW group.

**Results:**

Mortality before adulthood was 16.7 percentage points greater among individuals who were VP/VLBW compared with control infants born at term (95% CI 13.2-20.2). Among survivors, the likelihood of attaining less than secondary education was 4.3 percentage points greater (95% CI −0.8 to 9.4). Differences in economic activity and working hours were small and uncertain. Within the VP/VLBW group, each additional week of gestational age was associated with a 6.8 percentage point reduction in mortality (95% CI −12.7 to −1.0), with weaker associations for educational and labor market outcomes.

**Conclusions:**

VP/VLBW birth is associated with elevated mortality and educational disadvantage in early adulthood. These findings highlight the importance of long-term support for this population beyond neonatal survival, particularly in education and development policy.

The long-term consequences of preterm birth are becoming increasingly relevant as advances in neonatal care have significantly improved survival rates for infants born preterm.^1^^,^^2^ Although the short-term health challenges associated with preterm birth are well documented, there is limited understanding of how early-life disadvantages shape outcomes in adulthood. Exploring associations between very preterm (VP) birth and very low birth weight (VLBW) with later-life indicators such as educational attainment and labor market participation can offer important insights for health and social policy.

A key challenge in studying long-term outcomes is the lack of data following individuals born preterm into adulthood. Most studies focus on outcomes in childhood or adolescence, leaving a critical gap in our understanding of how early disadvantages evolve over the life course. This study addresses this limitation by using data from the RECAP Preterm Project,^3^, ^4^, ^5^, ^6^ which harmonizes multiple cohort studies of individuals born VP or VLBW across 5 high-income countries: Canada, Germany, the Netherlands, New Zealand, and Norway. These cohorts follow participants into early or midadulthood, enabling analysis of long-term educational and economic outcomes as well as mortality.

We adopt a 2-pronged methodological approach. First, we use coarsened exact matching (CEM) to estimate differences in adulthood outcomes between individuals who were VP/VLBW and control patients born at term, controlling for a range of covariates. Second, we assess how outcomes vary with each additional week of gestational age within the VP/VLBW group. To address potential endogeneity in gestational age, we used an instrumental variable (IV) strategy, using nulliparity as an instrument—a variable shown in previous literature to be associated with gestational duration.^7^, ^8^, ^9^, ^10^

By combining harmonized data from multiple countries with rigorous empirical methods, this study seeks to deepen understanding of how early gestational disadvantages translate into adulthood outcomes and whether these effects vary incrementally with gestational age. The findings contribute to evidence on the life-course consequences of preterm birth and inform the design of policies aimed at supporting this vulnerable population.

## Methods

### Study Population

Our data come from the RECAP Preterm Project.^3^^,^^5^^,^^6^ which is a consortium of longitudinal cohort studies of individuals born VP/VLBW in developed countries. Our analysis uses data from 5 countries: the Netherlands (Project on Preterm and Small for Gestational Age Infants [POPS]), Germany (data from Bavaria, Bavaria Longitudinal Study Cohort), New Zealand (NZ), Canada (CD) and Norway (Norwegian University of Science and Technology). We chose these countries because they have follow-up surveys in adulthood. Table I presents details of the birth year of each cohort, the number of adulthood follow-ups available, and the age of the cohorts at the time of these follow-ups. In addition, Table I outlines the inclusion criteria for each study and indicates whether a term-born control group is available for comparison.

**Table I:** Specification of data sets

Geographic regions	Birth year	Number of adulthood follow-ups	Age at follow up	Selection criteria	Control group
Netherlands (POPS)	1983	3	19, 28, 35	Gestational age <32 or BW <1500 g	Not available
Bavaria, Germany (Bavaria Longitudinal Study Cohort)	1985-1986	1	26-28	Gestational age <32 or BW <1500 g	Term born
New Zealand (NZ)	1986	2	22-23, 26-30	BW < 1500 g	Term born
Canada (CD)	1977-1982	2	24, 35	BW < 1000 g	Term born
Norway (Norwegian University of Science and Technology)	1986-1988	1	26	BW < 1500 g	Term born

All cohorts in our study were born in the 1980s, with the exception of the Canadian cohort, which is 1-8 years older than the median of other cohorts. For all 5 countries, we have follow-up data collected when the participants were in their mid-20s, which is the primary age range we focus on throughout this research. In terms of inclusion criteria, 4 countries included individuals on the basis of very low birth weight (birth weight [BW]< 1500 g) and/or VP birth (gestational age <32 weeks). However, for Canada, the selection criteria are stricter, focusing on extremely low birth weight (BW <1000) and extreme preterm birth (gestational age <28 weeks), respectively. We pooled data from all 5 countries to create a comprehensive dataset of 2493 individuals born very preterm and/or very low birth weight, referring to our combined sample as the VP/VLBW sample henceforth.

### Explanatory Variables

To account for potential confounding factors, we selected control variables that were available across all 5 cohort studies, including birth outcomes (BW, Hadlock small for gestational age [SGA] indicator,^11^ sex), parental characteristics (parental education), and neonatal outcomes (duration of assisted ventilation), all of which is measured at birth.

For parental characteristics, we used the education level of the least-educated parent as a key variable (we also tried education level of the mother, and the results were similar). To ensure consistency across all countries, we harmonized the years of education by constructing an indicator variable that equaled one if the least-educated parent had lower secondary education International Standard Classification of Education 0-2). Regarding neonatal outcomes, we included the duration of assisted ventilation as a measure of neonatal health status.

### Outcome Measures

The outcomes of our study were measured when the participants were in their mid-20s. We selected variables that are consistently available across all datasets. We began by examining the mortality rate before adulthood, because all individuals observed for educational or labor market outcomes are survivors. This means our analysis is inherently limited to those who represent the greater end of the distribution in terms of gestational length and/or BW, because individuals with poorer outcomes may not have survived to be observed.

### Statistical Analysis

Our analysis included 2 approaches. First, we examined the differences in adult outcomes between the VP/VLBW sample and the control group born at term. Individuals in the control group were selected from infants born at term (gestational age ≥37 weeks) in the same year. This approach focuses on comparing the levels of outcomes between our sample of interest and individuals with normal birth characteristics. Next, we concentrated on the VP/VLBW sample alone, investigating the impact of one additional week of gestational age on adulthood outcomes. This method allowed us to assess the marginal effect of gestational age within the VP/VLBW sample.

#### Matching Method

To achieve the first goal, we matched the observations using the CEM method,^12^ based on all baseline characteristics available in both the VP/VLBW sample and the term born control group, such as sex, family background, maternal age, nulliparity, and country (see Van Beek et al^13^ for similar choice of baseline variables). Because the Netherlands (POPS) cohort did not include a term-born control group, data from the Netherlands were excluded from the analysis in this section. In addition, New Zealand data do not have any information about mortality before adulthood in the control group. Hence, we have to exclude New Zealand from the mortality analysis in this subsection.

For family background, we relied on parental education level as a consistent variable available across all countries and in both the VP/VLBW and control groups. We present results using the CEM method as our baseline. We used the CEM function from the cem package in R with automatic cut-points. For robustness, we also report results using propensity score matching.^14^ We used the nearest neighbor method via the MatchIt package in R, with a logistic regression model to estimate propensity scores. Both methods are effective in evaluating the average treatment effect on treated individuals^15^; however, some studies suggest that CEM may perform better with smaller number of baseline covariates for matching.^16^, ^17^, ^18^

More specifically, we regressed the outcome variable of individual *i* (*Y*_*i*_) on a treatment indicator, which takes the value of 1 if the individual was VP/VLBW and 0 otherwise (treatment_*i*_), along with country fixed effects (*c*_*i*_) to control for country-specific differences in outcome levels. The regression model is specified as:

(1) $$Y_{i}=\alpha \;\cdot \;{\text{treatment}}_{i}+c_{i}+\epsilon_{i}$$

In this equation, *α* represents the average treatment effect on the treated individuals, accounting for country-specific fixed effects (*c*_*i*_). *ϵ*_*i*_ is the error term.

#### IV Method

Our second approach involved investigating the effects of 1 extra week of gestational age if the infant was born VP/VLBW. For this purpose, we limited our observations to the VP/VLBW sample and regressed the outcome variables we previously mentioned on gestational age, along with a set of control variables for birth outcomes, family background, and neonatal care received.

Specifically, in equation (2), we regressed the outcome variable for individual *i* (*Y*_*i*_) on the duration of gestational age (GA_i_), along with a set of control variables *X*_*i*_ and country fixed effects *c*_*i*_. For the control variables, we chose those that are commonly controlled for in the literature (see, eg, Medlock et al^19^) and included parental education and maternal age to represent family background, sex, an indicator for Hadlock measure of SGA,^11^ BW for birth outcomes, and the duration of assisted ventilation as a measure of neonatal health status. We used clustered SEs at country level to account for within country correlations (we used R package lfe to run the IV regression).^20^

(2) $$Y_{i}=\beta \;\cdot \;{\text{GA}}_{i}+{\;X}_{i}+{\;c}_{i}+\varepsilon_{i}$$

There is a potential bias in estimating equation (2) because not all relevant characteristics that influence both gestational age and later outcomes are observed in our data. Therefore, there may be omitted variables (eg, household income) that are correlated with both gestational age and the outcome variable *Y*_*i*_. To address this issue, we used an IV for gestational age that is correlated with gestational age but is independent of the error term in equation (2).

Our proposed IV is an indicator of whether the mother is nulliparous. Several studies have demonstrated that nulliparity is associated with lower gestational age and an increased risk of prematurity.^7^, ^8^, ^9^, ^10^ The Norwegian cohort was excluded from the IV analysis because information on maternal nulliparity was not available in this dataset.

## Results

### Testing the Model Hypothesis

#### Matching Method

Table II shows the balance of baseline variables before and after performing CEM. As shown, there were fewer girls and more parents with low education in the VP/VLBW group compared with the control group. In addition, mothers in the VP/VLBW group tended to be younger, and there were fewer nulliparous mothers. After performing the matching, we found that the differences between the 2 groups became smaller, and for most variables, the difference was no longer statistically significant, except for maternal age. However, it is worth noting that the difference in mean maternal age between the groups after matching was reduced to just 1 year, which mitigated the potential bias from this imbalance.

**Table II:** Balance of baseline variables before and after matching

Variables	VP/VLBW			Control			Diff	*P* value
	Mean	SD	No.	Mean	SD	No.		
Before matching								
Female	0.47	0.50	1157	0.54	0.50	595	−0.07	.01
Parent with low education	0.13	0.33	1157	0.08	0.28	595	0.04	.01
Mother’s age	27.10	5.45	1157	28.90	4.63	595	−1.80	<.01
Nulliparous	0.47	0.50	1157	0.53	0.50	595	−0.07	.04
After matching								
Female	0.50	0.50	758	0.49	0.50	510	0.02	.57
Parent with low education	0.07	0.25	758	0.06	0.24	510	0.00	.73
Mother’s age	27.70	4.83	758	28.80	4.55	510	−1.18	<.01
Nulliparous	0.50	0.50	758	0.54	0.50	510	−0.03	.32

The reported *P* values reflect the results of 2-sample *t* tests comparing the VP/VLBW and control groups.

#### IV Method

The IV method relies on 2 key assumptions: relevance, meaning the instrument must be strongly correlated with the endogenous variable, and exclusion restriction, which requires that the instrument—here, an indicator for nulliparity—affects the outcome (eg, educational attainment or labor market participation) only through gestational age. To assess relevance, we regressed gestational age on nulliparity. Table III, column 1, shows a strong correlation, with an F-statistic of 198, well above the threshold for weak instruments.^21^ Including covariates in column 2 reduced the correlation slightly, but the F-statistic remained greater than 29 (for a single endogenous variable and a single instrument, a first-stage F-statistic >25 indicates that the instrument is not considered weak^20^). We accounted for nonrandom attrition using inverse probability weighting; the first-stage regression with inverse probability weighting (column 3) yielded an even greater F-statistic, further confirming instrument strength.

**Table III:** First-stage regression of gestational age on nulliparity

Variables	Dependent variable: gestational age		
	(1)	(2)	(3)
Nulliparous	0.719 (0.051)	0.361 (0.066)	0.360 (0.063)
Observations	1636	1636	1636
Adjusted R^2^	0.184	0.694	0.692
Country FE	Yes	Yes	Yes
Controls	No	Yes	Yes
IPW	No	No	Yes
F-stat of excluded var.	198.75	29.91	32.65

*IPW*, inverse probability weighting.

Shown is the first stage regression of equation (2). In column 1 no control variable is added. In column 2, we add all sets of control variables. In column 3 we use IPW. Standard errors are clustered at country level. Norway is excluded from the analysis as it does not have any information about nulliparity. Control variables are: maternal age, parental low education indicator, sex, SGA, birth weight, duration of assisted ventilation.

The exclusion restriction cannot be directly tested, but we adopted 2 strategies to assess its plausibility. First, previous studies have shown seasonal variation in gestational age.^22^, ^23^, ^24^ In the New Zealand cohort, we used season of birth as an additional instrument and conducted a Sargan test^25^ to assess whether the instruments were valid. The test yielded a *P* value of .22, suggesting no evidence that the instruments were invalid or that the model violated the required statistical assumptions. Second, we tested whether nulliparity correlated with maternal characteristics such as smoking, socioeconomic status, marital status, and ethnicity, known to be associated with both neonatal outcomes and social determinants.^26^^,^^27^ We found no significant associations, supporting the validity of the exclusion restriction.

### Summary Statistics

Table IV presents the summary statistics for 4 groups of variables for the VP/VLBW and control groups. Please note that some variables are not available for some countries. For example, in the control group of Canada, we do not have information about maternal age. In, Norway, we do not have information about nulliparity neither in VP/VLBW nor in control group. Table IV only includes observations that have all birth outcome and parental characteristics measures in both VP/VLBW and control group. In our VP/VLBW cohort, the average gestational age was 30 weeks, and the average birth weight was 1179 g, which was approximately 10 weeks and 2200 g lower than control group, respectively. There were fewer female patients in our VP/VLBW sample. In addition, 43% of the VP/VLBW sample were SGA, which was a substantially greater proportion compared with the control cohort, where only 4.6% were SGA. Furthermore, one-half of the individuals who were VP/VLBW represented the first pregnancy of their mothers in both groups.

**Table IV:** Summary statistics of variables of the study

Variables	Mean	SD	No.	Mean	SD	No.	Diff	*P* value
Birth outcomes								
Gestation age, wk	29.60	2.93	2493	39.80	1.01	496	−10.20	*<*.01
Female, %	47.10	49.90	2489	53.80	49.90	595	−6.69	*<*.01
BW, %	1179.0	323	2493	3450	481	496	−2270	*<*.01
SGA, %	43.40	49.60	2489	4.64	21.00	496	38.76	*<*.01
Nulliparous, %	49.90	50.00	2176	53.30	50.00	330	−3.47	.24
Parental characteristics								
Parent with low education, %	27.10	44.50	1845	8.29	27.60	591	18.81	*<*.01
Mother's age, %	27.20	5.15	2416	28.90	4.63	445	−1.72	*<*.01
Neonatal outcomes								
Duration of assisted ventilation, d	12.70	28.00	2336	NA	NA	-	-	-
Adulthood outcomes								
Mortality rate, %	28.30	45.10	2493	0.81	8.95	496	27.49	*<*.01
Low education, %	24.90	43.30	987	16.90	37.50	539	8.00	*<*.01
Number of working hours	33.40	14.80	802	34.20	13.90	344	−0.80	.40
Economically active, %	78.70	40.90	1025	79.30	40.50	542	−0.60	.78

Mortality rate shows mortality at any time before 28 year survey which means 28.3% of 2493 live births died before reaching 28 years old. The number of observations differs between outcomes in the control cohort because not all countries provided mortality data for the control group (New Zealand lacked such data), whereas educational data were available for all cohorts in control group. Consequently, the number of observations for the education variable (n = 539) is slightly greater than for mortality (n = 496). POPS does not have any control group. Mean shows the average, SD shows the SD and No. shows the number of observations. The reported *P* values reflect the results of 2-sample *t* tests comparing the VP/VLBW and control groups. For binary variables (coded 0 and 100), the mean represents the percentage of individuals with the value 100, and the SD is calculated from this binary coding.

In terms of parental education, In our VP/VLBW sample, 27% of infants have parents with low education, which was significantly greater than the control sample by a 19 percentage point difference. Average maternal age was 27 years at the time of giving birth in the VP/VLBW sample, which was 2 years lower than in the control group. On average, infants in the VP/VLBW cohort received 13 days of assisted ventilation after birth.

In terms of outcomes, Table IV shows that approximately 28.3% of the VP/VLBW sample died before reaching adulthood. This is 35 times greater than the mortality rate for children born at term. We then considered 4 types of outcomes that are recorded in most of the cohorts. It is important to note that the number of observations decreases when analyzing follow-up surveys as the result of attrition across all cohorts. Strategies to account for this attrition are discussed at the beginning of the Method section.

We assessed the probability of having low education, defined as having less than secondary education (International Standard Classification of Education 0-2 levels), with approximately 25% of respondents classified as having low education in the VP/VLBW sample, which was 8 percentage points greater than for the controls. In addition, 80% of the VP/VLBW sample was economically active, meaning they were engaged in paid employment. Among those who were employed, the average working hours were 33 hours per week. This working hour information was available for all countries except Norway. We did not observe any significant difference in the mean of these 2 adulthood outcomes between the VP/VLBW and control groups.

### Comparing Outcomes between Patients Who Were VP/VLBW and Patients Born at Term

In this subsection, we compare the differences in the adulthood outcomes between the VP/VLBW sample and the control term-born group. It should be noted that for this analysis we included all countries except the Netherlands, which did not have a control group. The point estimate of the coefficient *α* of equation 1 is reported in Table V.

**Table V:** Coarsened exact matching between infants who were VP/VLBW and/or born at term

Outcome variables	Mortality	Low education	Economically active	Number of working hours
Treated	0.167 (0.018)	0.043 (0.026)	−0.035 (0.028)	0.43 (1.287)
Observations	1268	1268	1268	986
Country FE	Yes	Yes	Yes	Yes

*FE*, fixed effects.

The Netherlands was excluded from the analysis because it does not have a control cohort. New Zealand does not have mortality data for control cohorts and is omitted from the first column. SEs are reported in the parenthesis.

The probability that an individual born VP/VLBW died before adulthood was 16.7 (95% CI 13.2-20.2) percentage points greater compared with patients born at term. This considerable difference suggests that those who survive are a particularly select group within the VP/VLBW sample. Despite this, survivors still experience worse outcomes in adulthood in comparison with the control group. Specifically, they had a 4.3 (95% CI −0.8 to 9.4) percentage point greater probability of being low educated. We did not observe any statistically significant differences in terms of economic activity (95% CI −9 to 2, percentage point) and working hours (95% CI −2.092 to 2.952 hours); however, the signs of the point estimates are consistent with the scenario of worse adulthood outcomes for individuals born VP/VLBW.

We also used propensity score matching and compared results. Point estimates are similar in terms of the sign of the coefficient but they are different in size. For example, using the PSM method, the probability that an individual born VP/VLBW died before adulthood was 9.8 (95% CI 5.7-13.9) percentage points higher compared with control patients born at term and the probability of being low educated was 8 (95% CI 1.9- 14.1) percentage points greater.

### The Value of Extra Gestational Weeks for VP/VLBW

In the previous subsection, we found evidence of lower educational attainment among individuals who are VP/VLBW in adulthood, although this association was only significant at 10% CI. In this subsection, we focus on investigating how gestational age influences adulthood outcomes within the VP/VLBW sample.

Table VI displays the ordinary least squares estimates from equation 2. The findings show that each additional week of gestational age decreases the probability of death before reaching adulthood by 1.8 (95% CI −2.8 to −0.8) percentage points. We did not observe any statistically significant effect in terms of other adulthood outcomes.

**Table VI:** Ordinary least squares and two stage least square effect of 1 extra gestational week on the outcomes for VP/VLBW

Variables	Mortality	Low education	Economically active	Number of working hours
	(1)	(2)	(3)	(4)
Ordinary least squares				
Gestational age	*−*0.018 (0.005)	0.011 (0.011)	*−*0.005 (0.006)	0.292 (0.265)
Two stage least square				
Gestational age	−0.0682 (0.0299)	−0.168 (0.104)	−0.0862 (0.106)	0.749 (1.357)
Observations	1,636	887	918	761
Controls	Yes	Yes	Yes	Yes
Country FE	Yes	Yes	Yes	Yes
Mean of dep. var.	0.11	0.27	0.79	33.3

SEs are clustered at country level. Norway was excluded from the analysis because it does not have any information about nulliparity. Control variables are maternal age, parental low education indicator, sex, SGA, BW, and duration of assisted ventilation.

Table VI also presents the results of the IV analysis, in which maternal nulliparity was used as an instrument for gestational age. Compared with the ordinary least squares results, the estimated effect of gestational age became stronger after accounting for potential bias from unobserved factors. This suggests that infants born at lower gestational ages may also be more likely to have other unmeasured disadvantages, such as neonatal complications or less-favorable socioeconomic conditions, which influence later outcomes. We found that greater gestational age at birth significantly reduced the probability of mortality before adulthood by 6.8 (95% CI −12.7 to −1.0) percentage points, underscoring the role of additional gestational time in enhancing survival rates. However, we did not observe any statistically significant effects of gestational age on other adulthood outcomes, such as low educational attainment (95% CI −37.2 to 3.6 percentage points), economic activity (95% CI −29.4 to 12.2] percentage point), or working hours (95% CI −1.912 to 3.411 hours).

One potential concern is that the duration of assisted ventilation may be censored for infants who died shortly after birth, which could bias the estimated effect of gestational age on later outcomes. However, our 2SLS results remain robust when we exclude all control variables or omit only the assisted ventilation variable (see the Appendix; available at www.jpeds.com). SGA might also be multicollinear with BW and gestation age. We omitted SGA from the list of covariates and the results remained stable (Appendix; available at www.jpeds.com). Another concern is the nonlinearity in mortality with respect to gestational age. We tried probit and logit model for the mortality, and marginal effects are quite similar to the linear probability model.

## Discussion

This study contributes to the growing body of research on the life-course implications of VP/VLBW, highlighting substantial disparities in outcomes between individuals born VP/VLBW and their counterparts born at term. The observed mortality risk before adulthood was notably greater among the VP/VLBW group, even when limiting the sample to individuals discharged after birth. Among survivors, there was a greater likelihood of lower educational attainment. Although estimates for labor market participation and working hours did not show large effects, the direction of associations suggests potential long-term disadvantages in economic engagement.

Within the VP/VLBW group, we found that each additional week of gestation was associated with a marked reduction in the probability of mortality before adulthood, although the effect on educational and economic outcomes was less pronounced. These results are consistent with a gradient in vulnerability linked to gestational age, particularly with regard to survival, and point to the importance of early gestational development as a determinant of later outcomes.

Our findings are in line with earlier research from various European countries showing negative associations between preterm birth and educational achievement of school aged children.^28^, ^29^, ^30^, ^31^, ^32^ Several studies emphasize that the effects are strongest for individuals born extremely preterm (<28 weeks).^32^^,^^33^ Additional birth-related indicators—such as being SGA or having low Apgar scores—also have been linked to educational disadvantage in studies from Sweden and Denmark.^28^^,^^34^ However, there is less evidence on the persistence of these disadvantages into adulthood, largely as a result of limitations in available data.

Our study builds on emerging research tracking individuals born preterm into adulthood.^35^^,^^36^ A meta-analysis, for example, found that adults who were VP/VLBW were less likely to complete secondary education or be employed and more likely to receive welfare benefits compared with adults who were born at term.^37^ Our findings are broadly consistent with this pattern and strengthen the case for early developmental disadvantage persisting into adult life. Although we did not observe differences in all adulthood outcomes, this may reflect limited statistical power in later-life measures or delayed emergence of effects.

### Strengths and Limitations

Key strengths of this study include the use of harmonized, nationally representative cohort data from multiple high-income countries and the application of rigorous methods, including coarsened exact matching and an IV approach. The availability of a term-born comparison group in 4 cohorts enhances interpretability.

However, the study is not without limitations. First, longitudinal follow-up into adulthood resulted in some attrition, which may introduce bias. Second, the Dutch POPS cohort did not include a term-born control group, reducing comparability. Third, heterogeneity in data collection and definitions across cohorts constrained our ability to harmonies variables and limited the scope for alternative instruments in the IV analysis. Fourth, findings may not generalize to lower-income settings with different health care systems. Fifth, the absence of strong associations with labor market outcomes may reflect low statistical power, as the number of individuals who are VP/VLBW and who have reached adulthood remains modest. Sixth, the definition of adulthood in this study is limited to the mid-20s, whereas key transitions in education and employment may continue well into the 30s in contemporary contexts.^38^^,^^39^

Please note that our adulthood outcomes are measured in early adulthood, and as mentioned previously, many individuals in the control group are not low educated—that is, they are still in higher education. Therefore, finding no statistically significant effects on labor market outcomes is expected. These effects may become more pronounced in later adulthood. Finally, because the participants in the cohorts in this study were born in the 1980s, the results reflect obstetrical and neonatal care practices of that era. Consequently, these findings may not fully represent outcomes for contemporary cohorts of extremely preterm infants, who have benefited from substantial advances in perinatal and neonatal medicine.

### Implications for Policy and Practice

Our results underscore the need to address not only survival but also long-term developmental trajectories in individuals born VP. Although mortality in high-income settings has decreased as the result of advances in neonatal care,^40^ many individuals who were born VP/VLBW continue to face enduring cognitive, educational, and psychosocial challenges.^41^ Structured early intervention programs, including parental support, developmental therapy, and tailored educational services, could help mitigate these challenges. Schools also may play a critical role in supporting preterm-born children through individualized learning plans and better awareness of neurodevelopmental differences.

### Directions for Future Research

Further research should explore the mechanisms through which gestational age affects long-term outcomes and how these are shaped by postnatal interventions, family environment, and socioeconomic status. Longitudinal studies extending into later adulthood would help clarify whether the disadvantages seen in the mid-20s widen or diminish over time.

## Data Statement

Data sharing statement available at www.jpeds.com.

## CRediT authorship contribution statement

**Hanifa Pilvar:** Writing – review & editing, Writing – original draft, Visualization, Validation, Methodology, Investigation, Formal analysis, Conceptualization. **Catia Nicodemo:** Writing – review & editing, Supervision, Methodology, Funding acquisition, Conceptualization. **Stavros Petrou:** Writing – review & editing, Supervision, Funding acquisition, Conceptualization. **Brian A. Darlow:** Writing – review & editing. **Paula van Dommelen:** Writing – review & editing. **Kari Anne I Evensen:** Writing – review & editing. **Sarah Harris:** Writing – review & editing. **John Horwood:** Writing – review & editing. **Karen Mathewson:** Writing – review & editing. **Saroj Saigal:** Writing – review & editing. **Louis Schmidt:** Writing – review & editing. **Dieter Wolke:** Writing – review & editing. **Lianne J. Woodward:** Writing – review & editing. **Sungwook Kim:** Writing – review & editing, Supervision, Methodology, Funding acquisition, Conceptualization.

## Declaration of Competing Interest

The authors declare no conflict of interest.

## Supplementary data

Appendix

Data statement

## References

[1] Santhakumaran S., Statnikov Y., Gray D., Battersby C., Ashby D., Modi N. (2018). Survival of very preterm infants admitted to neonatal care in England 2008-2014: time trends and regional variation. Arch Dis Child Fetal Neonatal Ed.

[2] Butler V., Gaulard L., Sartorius V., Ancel P.Y., Goffinet F., Fresson J. (2024). Trends in the survival of very preterm infants between 2011 and 2020 in France. Arch Dis Child Fetal Neonatal Ed.

[3] Bamber D., Collins H.E., Powell C., Gonçalves G.C., Johnson S., Manktelow B. (2022). Development of a data classification system for preterm birth cohort studies: the RECAP preterm project. BMC Med Res Methodol.

[4] Darlow B.A., Horwood L.J., Woodward L.J., Elliott J.M., Troughton R.W., Elder M.J. (2015). The New Zealand 1986 very low birth weight cohort as young adults: mapping the road ahead. BMC Pediatr.

[5] Teixeira R., Queiroga A.C., Freitas A.I., Lorthe E., Santos A.C., Moreira C. (2021). Completeness of retention data and determinants of attrition in birth cohorts of very preterm infants: a systematic review. Front Pediatr.

[6] Teixeira R., Doetsch J., Freitas A.I., Lorthe E., Santos A.C., Barros H., RECAP Preterm Consortium (2022). Survey of data collection methods and retention strategies in European birth cohorts of children and adults born very preterm. Paediatr Perinat Epidemiol.

[7] James S., Gil K.M., Myers N.A., Stewart J. (2009). Effect of parity on gestational age at delivery in multiple gestation pregnancies. J Perinatol.

[8] Koullali B., Van Zijl M.D., Kazemier B.M., Oudijk M.A., Mol B.W.J., Pajkrt E. (2020). The association between parity and spontaneous preterm birth: a population based study. BMC Pregnancy Childbirth.

[9] Lin L., Lu C., Chen W., Li C., Guo V.Y. (2021). Parity and the risks of adverse birth outcomes: a retrospective study among Chinese. BMC Pregnancy Childbirth.

[10] Szyszka M., Rzońca E., Rychlewicz S., Baczek G., Ślezak D., Rzońca P. (2023). Association between parity and preterm birth—retrospective analysis from a single centre in Poland. Healthcare.

[11] Hadlock F.P., Harrist R.B., Martinez-Poyer J. (1991 Oct). In utero analysis of fetal growth: a sonographic weight standard. Radiology.

[12] Iacus S.M., King G., Porro G. (2011). Multivariate matching methods that are monotonic imbalance bounding. J Am Stat Assoc.

[13] Van Beek P.E., Leemhuis A.G., Abu-Hanna A., Pajkrt E., Aarnoudse-Moens C.S.H., Van Baar A.L. (2022). Preterm birth is associated with lower academic attainment at age 12 years: a matched cohort study by linkage of population-based datasets. J Pediatr.

[14] Austin P.C. (2011). An introduction to propensity score methods for reducing the effects of confounding in observational studies. Multivariate Behav Res.

[15] Fullerton B., Pöhlmann B., Krohn R., Adams J.L., Gerlach F.M., Erler A. (2016). The comparison of matching methods using different measures of balance: benefits and risks exemplified within a study to evaluate the effects of German disease management programs on long-term outcomes of patients with type 2 diabetes. Health Serv Res.

[16] Guy D., Karp I., Wilk P., Chin J., Rodrigues G. (2021). Propensity score matching versus coarsened exact matching in observational comparative effectiveness research. J Comp Eff Res.

[17] King G., Nielsen R. (2019). Why propensity scores should not be used for matching. Polit Anal.

[18] Ripollone J.E., Huybrechts K.F., Rothman K.J., Ferguson R.E., Franklin J.M. (2020). Evaluating the utility of coarsened exact matching for pharmacoepidemiology using real and simulated claims data. Am J Epidemiol.

[19] Medlock S., Ravelli A.C.J., Tamminga P., Mol B.W.M., Abu-Hanna A. (2011). Prediction of mortality in very premature infants: a systematic review of prediction models. PLoS One.

[20] Cameron A.C., Miller D.L. (2015). A practitioner's guide to cluster-robust inference. J Hum Resour.

[21] Stock J.H., Yogo M. (2002). National Bureau of Economic Research (NBER) working paper t0284.

[22] Bodnar L.M., Simhan H.N. (2008). The prevalence of preterm birth and season of conception. Paediatr Perinat Epidemiol.

[23] Darrow L.A., Strickland M.J., Klein M., Waller L.A., Flanders W.D., Correa A. (2009). Seasonality of birth and implications for temporal studies of preterm birth. Epidemiology.

[24] Hviid A., Laksafoss A., Hedley P., Lausten-Thomsen U., Hjalgrim H., Christiansen M. (2022). Assessment of seasonality and extremely preterm birth in Denmark. JAMA Netw Open.

[25] Sargan J.D. (1958). The estimation of economic relationships using instrumental variables. Econometrica.

[26] Ion R., López Bernal A. (2015). Smoking and preterm birth. Reprod Sci.

[27] Roustaei Z., Räisänen S., Gissler M., Heinonen S. (2020). Associations between maternal age and socioeconomic status with smoking during the second and third trimesters of pregnancy: a register-based study of 932,671 women in Finland from 2000 to 2015. BMJ Open.

[28] Abel K., Heuvelman H., Wicks S., Rai D., Emsley R., Gardner R. (2017). Gestational age at birth and academic performance: population-based cohort study. Int J Epidemiol.

[29] Berbis J., Einaudi M.A., Simeoni M.C., Brévaut-Malaty V., Auquier P., d'Ercole C. (2012). Quality of life of early school-age French children born preterm: a cohort study. Eur J Obstet Gynecol Reprod Biol.

[30] Bhutta A.T., Cleves M.A., Casey P.H., Cradock M.M., Anand K.J.S. (2002). Cognitive and behavioral outcomes of school-aged children who were born preterm: a meta-analysis. JAMA.

[31] Eves R., Mendonça M., Baumann N., Ni Y., Darlow B.A., Horwood J. (2021). Association of very preterm birth or very low birth weight with intelligence in adulthood: an individual participant data meta-analysis. JAMA Pediatr.

[32] Ream M.A., Lehwald L. (2018). Neurologic consequences of preterm birth. Curr Neurol Neurosci Rep.

[33] Ahlsson F., Kaijser M., Adami J., Lundgren M., Palme M. (2015). School performance after preterm birth. Epidemiology.

[34] Ladelund A.K., Bruun F.J., Slavensky J.A., Ladelund S., Kesmodel U.S. (2022). Association of Apgar score at 5 minutes with academic performance and intelligence in youth: a cohort study. Acta Obstet Gynecol Scand.

[35] Johnson R.C., Schoeni R.F. (2011). The influence of early-life events on human capital, health status, and labour market outcomes over the life course. BE J Econ Anal Policy.

[36] Bilgin A., Mendonça M., Wolke D. (2018). Preterm birth/low birth weight and markers reflective of wealth in adulthood: a meta-analysis. Pediatrics.

[37] Baranowska-Rataj A., Barclay K., Costa-Font J., Myrskylä M., Özcan B. (2023). Preterm birth and educational disadvantage: heterogeneous effects. Popul Stud.

[38] Arnett J.J. (2000). Emerging adulthood: a theory of development from the late teens through the twenties. Am Psychol.

[39] Saigal S., Day K.L., Van Lieshout R.J., Schmidt L.A., Morrison K.M., Boyle M.H. (2016). Health, wealth, social integration, and sexuality of extremely low-birth-weight prematurely born adults in the fourth decade of life. JAMA Pediatr.

[40] Johnson S., Marlow N. (2017). Early and long-term outcome of infants born extremely preterm. Arch Dis Child.

[41] Wolke D., Johnson S., Mendonça M. (2019). The life course consequences of very preterm birth. Annu Rev Dev Psychol.

