# Supplementary material for: Outcomes in Early Adulthood for Individuals Born Very Preterm and/or with Very Low Birth Weight: Evidence from Multinational Cohorts
Source: J Pediatr Clin Pract. 2025 Dec 1;19:200196. doi: 10.1016/j.jpedcp.2025.200196 (PMC12810559; doi:10.1016/j.jpedcp.2025.200196)
Supplement: Appendix [file mmc1.docx]

# Appendix

## Inverse probability weighting

In our analysis, we created Inverse Probability Weights (IPWs) to adjust for non- random attrition in outcome variables in adulthood surveys, which is related to the education level of parents. We first generated a binary indicator (responded) to capture whether a respondent has missing data on the outcome variables. Then, we estimated the probability of response using a logistic regression, where the outcome is the responded variable, and the predictor is the education level of the parents. This model provides predicted probabilities (predict) of response for each observation. Finally, we calculated the IPWs by taking the inverse of the predicted probability, with the formula: *weight* =$\frac{1+\exp\left( predict \right)}{exp(predict)}$, which accounts for the likelihood of response based on the predictor variable. These weights will be used in subsequent regression models to correct for bias due to the non-random attrition.

## Extra tables

Table A1: Two SLS effect of one extra gestational week on the outcomes for VP/VLBW (without any control variable and without assisted ventilation)

|  | | Mortality | Low education | Economically active | Number of Working hours. |
| --- | --- | --- | --- | --- | --- |
|  |  | (1) | (2) | (3) | (4) |
| **Panel A: Two SLS without controls** | | | |  |  |
| GA | | *−*0.043^∗∗∗^ | -0.033 | *−*0.026 | 0.903 |
|  | | (0.014) | (0.025) | (0.053) | (1.002) |
| Observations | | 2,176 | 932 | 970 | 800 |
| Controls | | No | No | No | No |
| Country FE | | Yes | Yes | Yes | Yes |
| **Panel B: Two SLS without assisted ventilation in the covariates** | | | | | |
| GA |  | -0.064^∗∗^ | -0.147 | -0.060 | 1.209 |
|  |  | (0.031) | (0.112) | (0.107) | (1.518) |
| Observations |  | 1,701 | 905 | 940 | 779 |
| Controls |  | Yes | Yes | Yes | Yes |
| Country FE |  | Yes | Yes | Yes | Yes |
| **Panel C: Two SLS without SGA in the covariates** | | | | | |
| GA |  | -0.049^∗∗^ | -0.081 | -0.032 | 0.315 |
|  |  | (0.015) | (0.062) | (0.056) | (0.878) |
| Observations |  | 1,636 | 887 | 918 | 761 |
| Controls |  | Yes | Yes | Yes | Yes |
| Country FE |  | Yes | Yes | Yes | Yes |
| Mean of dep. | var. | 0.11 | 0.27 | 0.79 | 33.3 |

Note: Standard errors are clustered at country level. Norway is excluded from the analysis as it does not have any information about nulliparity. Control variables are: maternal age, parental low education indicator, sex, SGA, birth weight. ^∗^p*<*0.1; ^∗∗^p*<*0.05; ^∗∗∗^p*<*0.01
