# Supplementary material for: Outcomes in Early Adulthood for Individuals Born Very Preterm and/or with Very Low Birth Weight: Evidence from Multinational Cohorts
Source: J Pediatr Clin Pract. 2025 Dec 1;19:200196. doi: 10.1016/j.jpedcp.2025.200196 (PMC12810559; doi:10.1016/j.jpedcp.2025.200196)
Supplement: Data statement [file mmc2.docx]

Data Statement

The data used in this study were provided confidentially for this project and are not publicly available because of privacy and ethical restrictions. However, data can be requested from the RECAP Preterm Consortium, subject to approval and compliance with the consortium's data access policies.
